# Fusion between *Leishmania amazonensis* and *Leishmania major* Parasitophorous Vacuoles: Live Imaging of Coinfected Macrophages

**DOI:** 10.1371/journal.pntd.0000905

**Published:** 2010-12-07

**Authors:** Fernando Real, Renato A. Mortara, Michel Rabinovitch

**Affiliations:** Department of Microbiology, Immunology and Parasitology, Escola Paulista de Medicina, Universidade Federal de São Paulo (UNIFESP-EPM), São Paulo, Brazil; University of Queensland, Australia

## Abstract

Protozoan parasites of the genus *Leishmania* alternate between flagellated, elongated extracellular promastigotes found in insect vectors, and round-shaped amastigotes enclosed in phagolysosome-like Parasitophorous Vacuoles (PVs) of infected mammalian host cells. *Leishmania amazonensis* amastigotes occupy large PVs which may contain many parasites; in contrast, single amastigotes of *Leishmania major* lodge in small, tight PVs, which undergo fission as parasites divide. To determine if PVs of these *Leishmania* species can fuse with each other, mouse macrophages in culture were infected with non-fluorescent *L. amazonensis* amastigotes and, 48 h later, superinfected with fluorescent *L. major* amastigotes or promastigotes. Fusion was investigated by time-lapse image acquisition of living cells and inferred from the colocalization of parasites of the two species in the same PVs. Survival, multiplication and differentiation of parasites that did or did not share the same vacuoles were also investigated. Fusion of PVs containing *L. amazonensis* and *L. major* amastigotes was not found. However, PVs containing *L. major* promastigotes did fuse with pre-established *L. amazonensis* PVs. In these chimeric vacuoles, *L. major* promastigotes remained motile and multiplied, but did not differentiate into amastigotes. In contrast, in doubly infected cells, within their own, unfused PVs metacyclic-enriched *L. major* promastigotes, but not log phase promastigotes - which were destroyed - differentiated into proliferating amastigotes. The results indicate that PVs, presumably customized by *L. major* amastigotes or promastigotes, differ in their ability to fuse with *L. amazonensis* PVs. Additionally, a species-specific PV was required for *L. major* destruction or differentiation – a requirement for which mechanisms remain unknown. The observations reported in this paper should be useful in further studies of the interactions between PVs to different species of *Leishmania* parasites, and of the mechanisms involved in the recognition and fusion of PVs.

## Author Summary

Many non-viral intracellular pathogens lodge within cell vesicles known as “parasitophorous vacuoles” (PVs), which exhibit a variety of pathogen-dependent functional and compositional phenotypes. PVs of the protozoan Leishmania are similar to the digestive organelles known as phagolysosomes. We asked if, in phagocytes infected with two different Leishmania species, would the two parasites be found in the same or in separate vacuoles? Of the species chosen, *Leishmania amazonensis* develops within large vacuoles which shelter many parasites; in contrast, *Leishmania major* lodges in small PVs containing one or two parasites. In the present experiments, the species and their life-cycle stages (extracellular promastigotes, and intracellular amastigotes) were distinguished by means of fluorescent markers, and the intracellular localization of the parasites was examined in living cells. We report here that, whereas *L. major* amastigotes remained within their individual vacuoles, *L. major* promastigotes were delivered to *L. amazonensis* vacuoles, in which they survived and multiplied but were unable to differentiate into amastigotes. A species-specific vacuole was thus required for *L. major* differentiation. The model should be useful in cellular and molecular studies of the biology of these parasites and of their parasitophorous vacuoles.

## Introduction

In a classic review of intracellular parasitism, James Moulder proposed that microbial parasites customize the morphology, composition and function of parasitophorous vacuoles (PVs) in which they are sheltered [1]. Considering the implications of Moulder's proposal we asked if pathogens could survive and multiply within PVs that sheltered a different organism. In some instances it was possible to generate chimeric vacuoles in cells coinfected with different pathogens [2], [3], [4], [5]. In the present studies macrophages were coinfected with two species of *Leishmania* parasites normally lodged in PVs that differ in their biogenesis, morphology and parasite occupancy.

*Leishmania* are dimorphic trypanosomatid parasites, which induce cutaneous, muco-cutaneous or visceral disease in man and other animals. Elongated, proliferating, extracellular procyclic promastigote forms colonize the midgut of sandfly vectors. These forms, which can be grown axenically, differentiate into infective, stationary phase metacyclics promastigotes that can be released into the dermis of mammalian hosts in the course of the insect bloodmeal. Macrophages and other mammalian cells internalize infective promastigotes within PVs, in which parasites differentiate into the smaller, internally flagellated, oval-shaped amastigote forms. Amastigotes divide intracellularly and spread the infection in the mammal host [6].

*Leishmania* PVs are bound by a membrane - initially derived from the host cell plasma membrane - which undergoes compositional changes as they fuse with late endosomes/lysosomes and possibly with other vesicles. The phagolysosome-like nature of *Leishmania* PVs, initially supported by the acquisition of electron dense colloids by fusion of parasite-containing phagosomes with vesicles carrying the markers [7], [8], [9], [10], and by the demonstration that PVs were acidified, was reinforced by the detection of lysosomal markers such as lysosome-associated membrane proteins (LAMPs) and Rab GTPases in the PV membranes of a few *Leishmania* species examined [11], [12], [13]. Thus *Leishmania* PVs are considered acidic organelles, contain lysosomal enzymes and present a vacuolar pH in the range of 4.7–5.2 [12], [14].

Most studies on *Leishmania* PVs were performed with parasites of the *mexicana* group - *L. amazonensis* and *L. mexicana* – both sheltered in spacious PVs that may contain many amastigotes. These large, communal PVs were shown to selectively fuse with phagosomes containing large particles or microorganisms [15], [16], [17], [18]. Time-lapse microcinematographic studies revealed that most incoming zymosan-containing phagosomes remained in contact with *L. amazonensis* PVs for several hours before fusion took place, which itself lasted for only a few minutes [19]. More recently, fusion between *L. amazonensis* PVs was examined in macrophage cultures infected with amastigotes for 48 hours and reinfected with labeled amastigotes or promastigotes of the same species. Two hours after reinfection, the initially tight incoming PVs contacted the large recipient PVs; however, while both vacuoles stained positively for LAMP1 markers, fusion was only detected by 12 hours of reinfection, when both vacuoles were spacious. Thus, both labeled amastigotes or promastigotes could be transferred to large PVs which sheltered the same parasite species [20].

Most of *Leishmania* species studied, however, are lodged in small, membrane-bound PVs which display lysosomal markers, usually contain a single parasite and undergo fission as parasites divide [21], [22], [23]. It was reported that the pH within *L. donovani* membrane-bound PV is about 5.5, and the increase in vacuolar pH to 5.8 was not only tolerated by the parasites but exacerbated intracellular infection [24].

In the present studies, macrophages infected with *L. amazonensis* were challenged with *L. major* lesion amastigotes or promastigotes and coinfected cells observed by multidimensional live imaging. We found that whereas *L. major* amastigotes were excluded, *L. major* promastigotes were delivered into *L. amazonensis* PVs where they survived and multiplied but did not differentiate into amastigote forms.

## Materials and Methods

### Ethics statement

All experiments involving animal work were conducted under guidelines approved by UNIFESP and Institut Pasteur ethics committees, which are in accordance with international recommendations.

### Mice and parasites

BALB/c female mice, 8 weeks of age, were used as source of bone marrow cells. BALB/c nude mice, 8 weeks of age, were used as source of lesion-derived amastigotes after 2 months of the inoculum of wild-type *L. (L.) amazonensis* LV79 (MPRO/BR/72/M1841), or DsRed2-transfected *L. (L.) major* NIH173 (MHOM/IR/-/173) on mice footpad. Isolation of amastigotes from footpad lesions was performed as described previously [19].

*Leishmania major*-DsRed2 or GFP-transfected (MRHO/SU/59/P) promastigotes were cultivated at 26°C in an air atmosphere in M199 medium containing 10% fetal calf serum, 100 u/ml of penicillin, 100 µg/ml of streptomycin, and buffered with 10 mM HEPES at pH 7.2. Metacyclic enriched promastigote populations were separated in Ficoll PM type 400 gradients (Sigma-Aldrich Co.), as described elsewhere [25].

In growth studies *Leishmania major*-DsRed2 log phase promastigotes were seeded at 10^5^ parasites per well in 96 wells plates. Plates were cultivated at 34°C or 26°C in air atmosphere. For growth at pH 5.0 the buffer used was 2-morpholinoethanesulfonic acid (MES) at 10 mM. Wells were examined under an Olympus IX70 inverted microscope (10 x, 0.3 NA, 20 x, 0.4 NA, and 40 x, 0.6 NA objectives) equipped with an Olympus DP71 CCD Camera. Numbers of parasites per field were estimated from randomly acquired images of 10 microscopic fields in each of 3 wells, over periods of up to 10 days. Images were analyzed with Image Pro Plus 6 software (Media Cybernetics Inc.) for algorithm-based quantification. Parasite numbers were corrected to a 10 x objective field area.

### Infection of macrophage cultures

Bone marrow-derived macrophages were obtained and cultivated for 7 days in RPMI 1640 medium with 10% fetal calf serum, 5% L929 cell conditioned medium, 100 u/ml of penicillin and 100 µg/ml of streptomycin (complete medium) [26]. Macrophages were replated on round dishes (ibidi, GmbH), suitable for maintenance in incubators coupled to microscopes, or on 13 mm diameter coverslips placed in the wells of tissue culture plates. Before their use for experiments, cultures were kept overnight at 37°C, 5% CO_2_ in a humidified air atmosphere.

Lesion-derived amastigotes, stationary phase metacyclic-enriched promastigotes or log phase procyclic promastigotes were added to macrophages cultures at a multiplicity of infection of 5∶1 parasites to cell and incubated at 34°C, 5% CO_2_ in complete medium for different time periods according to the parasite stages used. Cultures were washed with Hanks' Buffered Salt Solution (HBSS) to remove free parasites, and cultivated in complete medium, 34°C, 5% CO_2_ in air atmosphere. To reduce the proliferation of extracellular promastigotes, cultures were washed and their medium replaced daily.

### Design of the experiments

In all experiments macrophage cultures infected for 48 hours with *L. amazonensis*-WT amastigotes were superinfected with *L. major*-DsRed2 amastigotes, metacyclic-enriched promastigotes or procyclic promastigotes. The first infection allowed for the development of large recipient PVs, to be distinguished from the small donor PVs that sheltered *L. major* parasites. Vacuoles containing parasites of the two species were denominated chimeric PVs. After different periods of superinfection, cultures were used for live imaging or fixed for immunolocalization. Superinfected cultures were examined to determine i) the occurrence of fusion between *L. amazonensis* and *L. major* PVs as a function of the stages of the later; ii) the proportion of the *L. major* intracellular parasites that were sheltered in chimeric vacuoles; iii) the proliferation and eventual differentiation of *L. major* parasites in superinfected compared to that in monoinfected macrophage cultures.

### Immunolocalization

Macrophages on coverslips were washed and fixed for 1 hour with 3.5% formaldehyde in phosphate buffered saline (PBS). Parasitophorous vacuoles and other acidic compartments were identified by immunolabeling of membrane proteins LAMP1 and LAMP2, with rat anti-mouse specific antibodies. *Leishmania amazonensis* amastigotes were identified and distinguished from *L. major* with the help of the 2A3-26 antibody conjugated to FITC (kindly provided by Dr. Eric Prina, Institut Pasteur, France). Cultures were then stained for 15 minutes with 100 µg/ml 4′,6-diamidino-2-phenylindole (DAPI) and mounted with 50% glycerol in PBS, containing 0.01% p-phenylenediamine. Confocal images were obtained with a Bio-Rad 1024UV system, coupled to a Zeiss Axiovert 100 microscope. Images acquired with a 100 x (1.4 NA) oil immersion objective were renderized by Imaris Software (Bitplane AG) using blend or MIP filters.

### Live imaging

Live imaging of cultures was performed by a Nikon Biostation IM Live cell recorder system (Nikon Corporation) and a Perkin-Elmer UltraView RS Nipkow-disk system (PerkinElmer Inc.) attached to a Zeiss Axiovert 200 M microscope with CCD detector Hamamatsu ORCA II ER. To identify *Leishmania* PVs, Lysotracker green DND-26 (Invitrogen Corporation), a lysosomotropic probe for acidic compartments, was added to complete medium at 10 µM concentration throughout image acquisition. Cultures were maintained at 34°C and 5% CO_2_, by incubators coupled to microscopes.

#### Conventional time-lapse acquisition

The Nikon Biostation IMq was used to acquire, in 10 different microscopic fields, serial images of superinfected macrophage cultures in multi-chamber dishes. The Biostation acquired images in phase contrast and in two fluorescent channels (for Lysotracker and DsRed2 labeled parasites), with 40 x objectives (0.8 NA) at intervals of 5 minutes. Time after superinfection is displayed as day-hours:minutes (dhh:mm).

Fluorescent parasites were quantified with Acapella software (Version 2.0 -PerkinElmer Inc.), which recognizes fluorescent patterns by algorithm-based image analysis.

#### Multidimensional acquisition

The Perkin-Elmer UltraView RS system was used to acquire approximately 20 focal stacks of 2 or more fields of live, superinfected macrophage cultures. Preparations were scanned at intervals of 15 minutes under 63 x (1.3 NA) oil objectives, to minimize phototoxicity and photobleaching of Lysotracker. Time after superinfection is displayed as hours:minutes (hh:mm).

Acquired images were processed by Imaris software (Bitplane AG) for construction of multidimensional images, comprising 3D, time and fluorescent channels. Surface rendering was used to measure parasites' volume, and sphericity (a parameter ranging from non-spherical 0 to spherical 1). Renderization using blend or MIP filters was used to visualize interaction between *Leishmania* PVs.

### Statistics

All experiments were repeated at least twice, with duplicate or triplicate coverslips in the case of fixed samples. Results presented in the form of images are representative of at least 2 other images analyzed. Statistical analyses (ANOVA) and graphs were built using SPSS 15.0 software (SPSS Inc.). Graphs display means and standard errors (s.e.m).

## Results

### Parasitophorous vacuoles that shelter *L. major* amastigotes did not fuse with *L. amazonensis* PVs

To determine if *L. amazonensis* and *L. major* PVs could fuse with each other, macrophages infected for 48 hours with *L. amazonensis* amastigotes were superinfected with *L. major*-DsRed2 amastigotes. Live cultures were loaded with Lysotracker to identify *Leishmania* PVs. Both live and fixed cultures were scanned to search for chimeric PVs.

**Figure 1:**
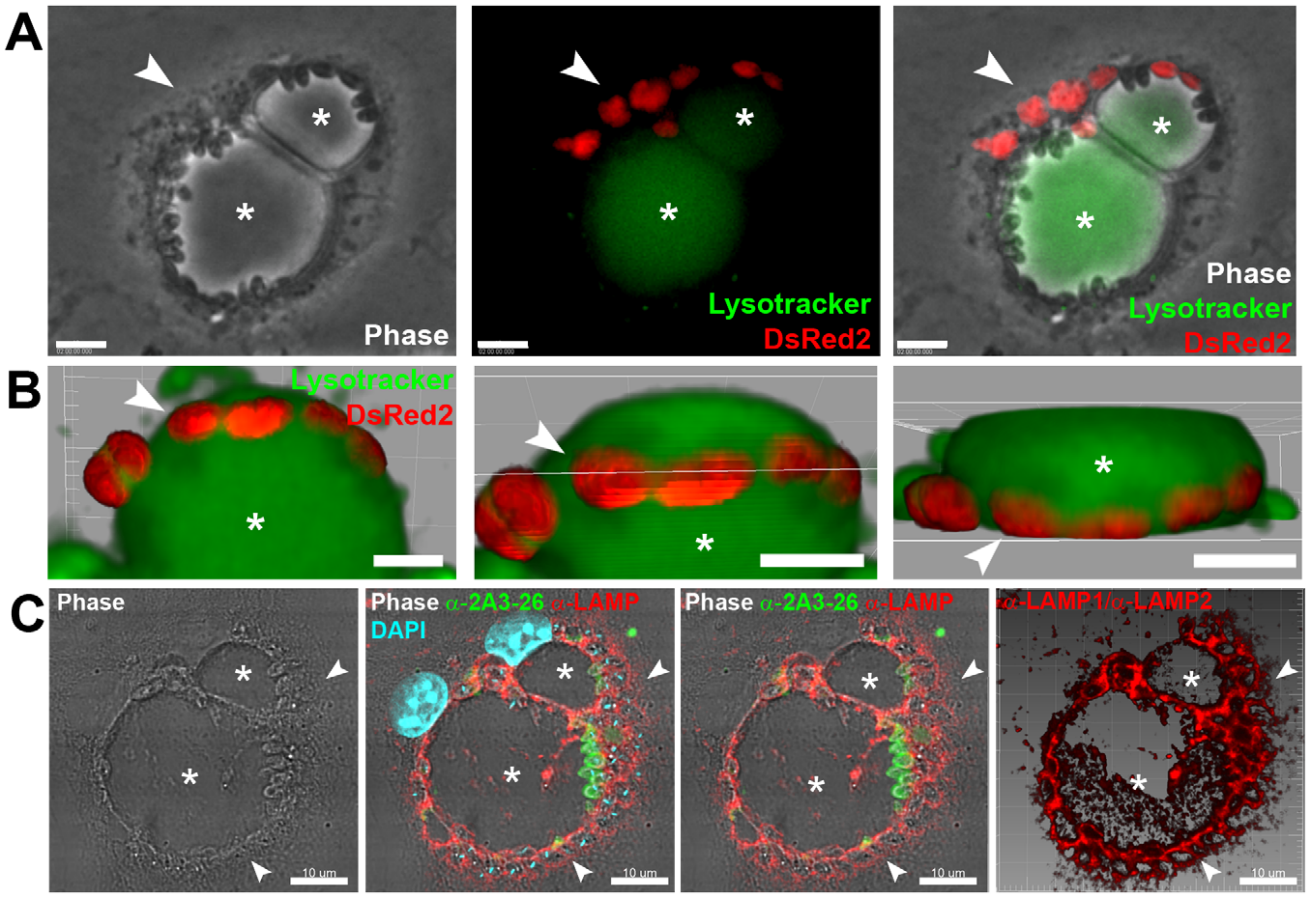
*L. major* amastigote PVs did not fuse with *L. amazonensis* vacuoles. (A) Macrophages were previously infected for 48 h with *L.* amazonensis-WT and then superinfected with *L. major*-DsRed2 amastigotes for additional 72 hours. Image shows a macrophage loaded with Lysotracker (green) and hosting the two parasite species under phase contrast channel (Ph2), fluorescence channels (Lysotracker and DsRed2), and merged channels, respectively. Asterisk indicates *L. amazonensis* PV and arrowheads indicate *L. major*-DsRed2 amastigotes (red). Bars = 10 µm. (B) Live multidimensional imaging of coinfected macrophages. Asterisk indicates *L. amazonensis*-WT PV stained with Lysotracker (green), surrounded by membrane-bound PVs with weak Lysotracker signal which shelter *L. major*-DsRed2 amastigotes (red). Multidimensional images were constructed by Imaris blend filter and each image represents a rotation of approximately 45°. Bars = 5 µm. (C) Immunolocalization of LAMP1/LAMP2 proteins in superinfected macrophages. *Leishmania major* amastigotes were sheltered by tight LAMP1/LAMP2-positive PVs (arrowheads), close to large recipient *L. amazonensis* PVs, indicated by asterisks. Image was acquired 11 days after *L. major*-DsRed2 amastigote addition. LAMP immunolabeling in red, 2A3-26 antibody (specific for *L. amazonensis* amastigotes) immunolabeling in green, DAPI staining in blue. Images are disposed as phase contrast (Ph3), phase contrast with RGB fluorescence channels, phase contrast with RG channels and 3D reconstruction of red channel with Imaris blend filter. Bars = 10 µm.

A few hours after superinfection, *L. major* PVs were found closely apposed to *L. amazonensis* PVs. However, for up to 11 days after superinfection, fusion between *L. major* and *L. amazonensis* PVs was not directly observed or inferred from finding of chimeric PVs (Fig. 1 and Video S1). In live cell recordings, the *L. major* PV is not visible since it did not take up detectable Lysotracker amounts possibly due to the small vacuolar volume between parasite and PV membranes. The possibility that the intravacuolar environment was less acidic cannot, however, be discarded.

Images that initially suggested fusion were later shown by multidimensional live imaging to result from *L. major* PVs positioned underneath *L. amazonensis* vacuoles (Fig. 1B and Video S1). Segregation of the two species of amastigotes in separate PVs was also confirmed by LAMP1/LAMP2 immunolabeling of superinfected macrophages (Fig. 1C). Similar results were obtained when macrophages were simultaneously infected with *L. amazonensis* and *L. major* amastigotes (data not shown). For up to 6 days after superinfection, *L. major* amastigotes, sheltered in individual PVs, multiplied at similar rates in monoinfected and superinfected phagocytes. These conclusions were supported by the observation of fixed cell preparations (data not shown).

### *Leishmania major* promastigotes were delivered to *L. amazonensis* vacuoles through PV fusion

**Figure 2:**
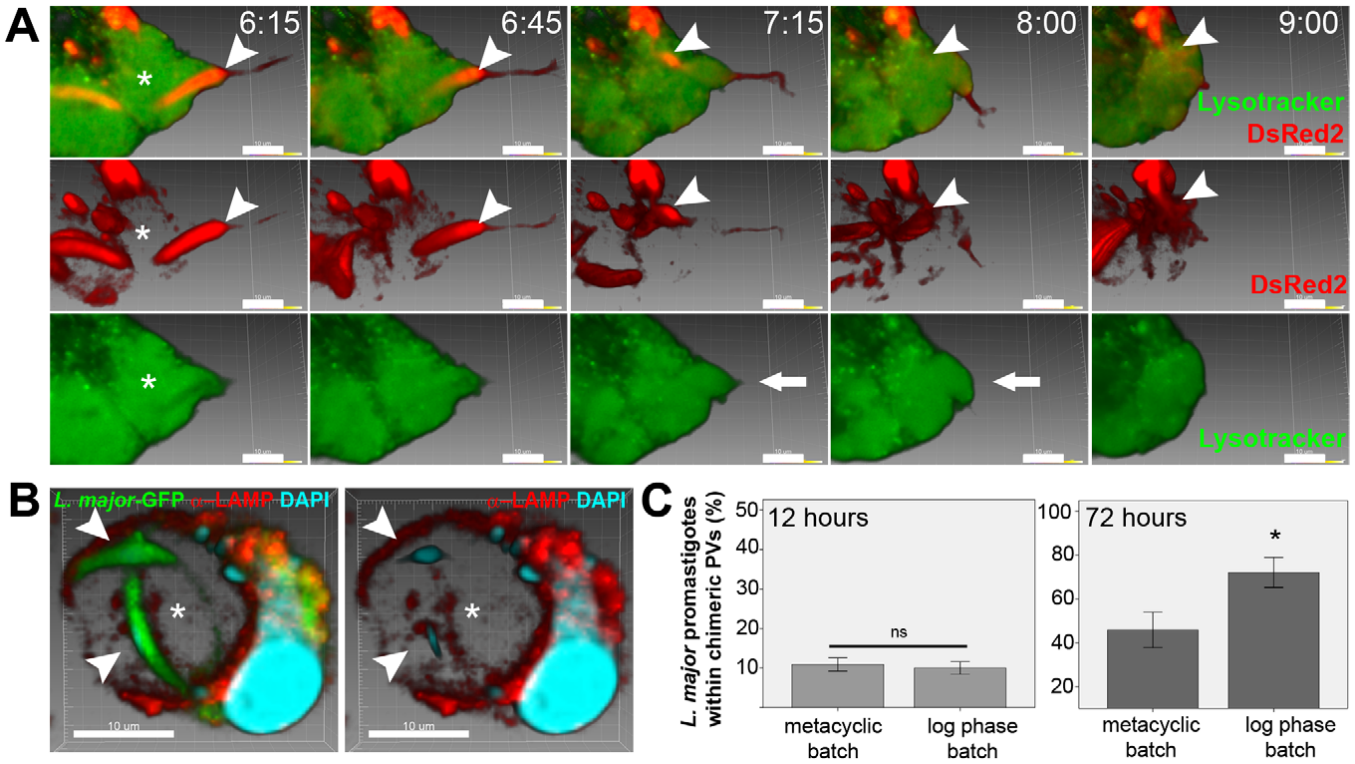
Fusion of *L. amazonensis* vacuoles with PVs that shelter *L. major* promastigotes. (A) Multidimensional imaging of macrophages infected with *L.* amazonensis-WT for previous 48 hours and superinfected with *L. major*-DsRed2 metacyclic-enriched promastigotes. Arrowhead indicates *L. major*-DsRed2 promastigote sheltered by tight PV, weakly stained with Lysotracker (green), interacting with large, Lysotracker-positive, *L. amazonensis*-WT PV (asterisk). In the first row, merged images of Lysotracker and DsRed2 signals show transfer of *L. major*-DsRed2 promastigote to *L. amazonensis* PV; time after promastigote addition is shown (h:mm). The second row shows DsRed2 signal, evidencing the transfer of promastigote by parasite posterior pole. The third row shows Lysotracker signal, showing changes in PV shape (bold arrow) to accommodate the incoming promastigote. Images were constructed using Imaris blend filter. Bars = 10 µm. (B) Immunolocalization of LAMP1 in superinfected macrophages; *Leishmania major*–GFP procyclic promastigotes (arrowheads) were sheltered by LAMP1-positive chimeric PV (asterisk). Image was acquired 48 h after *L. major*-GFP promastigote addition. LAMP1 immunolabeling in red, GFP in green, DAPI staining in blue. Three dimensional images, constructed by Imaris blend filter, are disposed as merged RGB fluorescence channels, merged RB channels and red channel. Bars = 10 µm. (C) Percentage of *L. major*-GFP promastigotes within chimeric PVs in fixed, superinfected macrophages. Samples were fixed 12 and 72 hours after addition of *L. major* promastigotes from metacyclic-enriched or log phase parasite batches. Columns are representative of 10 microscopic fields (under 100x objective) in triplicate samples. There is no statistical difference in the percentage of procyclic or metacyclic-enriched promastigotes within chimeric PVs at 12 hours post-superinfection. At 72 hours, a higher percentage of promastigotes from log phase superinfection batch within chimeric PVs was observed, comparing to superinfection with metacyclic-enriched batches (Univariate ANOVA, p<0.05).

In these experiments, macrophages infected for 48 hours with *L. amazonensis* were challenged with *L. major*-DsRed2 metacyclic-enriched promastigotes. In the first hours of superinfection, *L. major* donor vacuoles were found in video recordings and in fixed preparations to be in contact with *L. amazonensis* PVs. The contact regions were monitored by live multidimensional imaging. The sequence shown in Fig. 2A and Video S2 begins with the image of an *L. major* promastigote apparently apposed to an *L. amazonensis* PV loaded with Lysotracker.

In the early frames of the recording, the *L. major* promastigote occupied a PV which displayed a weak Lysotracker signal, contrasting with the stronger signal detected in the recipient *L. amazonensis* PV (Video S2). In subsequent images, the increase in volume occupied by the Lysotracker confirmed that the recipient PV was reshaped in the course of fusion with the *L. major* promastigote PV (Fig. 2A, bold arrow). The recording also shows that the posterior pole of the *L. major* promastigote was the first to be transferred to the recipient PV, in the opposite direction to the movement displayed by free *Leishmania* promastigotes. The duration of the fusion in this sequence was estimated to be around 80 minutes. The completion of fusion was heralded by the motility of the *L. major* promastigote within the *L. amazonensis* PV (Video S2).

Immunolabeling of LAMP1 proteins displayed by *L. amazonensis* PVs confirmed the existence of chimeric vacuoles containing *L. major* promastigotes from superinfection batches of procyclic (Fig. 2B) or metacyclic-enriched parasites (data not shown).

Delivery of *L. major* promastigotes to *L. amazonensis* PVs did not depend on metacyclogenesis, as approximately 10% of promastigotes (from logarithmic phase cultures or metacyclic-enriched baths of promastigotes) developed chimeric PVs in the first 12 hours of superinfection (Fig. 2C). After 72 hours of superinfection, 45.8% (±8.0 s.e.m., n = 3) of promastigotes from metacyclic-enriched batch were found within chimeric PVs as undifferentiated promastigotes; in these samples, we observed *L. major* amastigotes hosted within unfused donor vacuoles. At the same period after superinfection, 72% (±6.8 s.e.m., n = 3) of promastigotes from log phase batch were found within chimeric PVs, conserving promastigote morphology.

### *Leishmania major* promastigotes increase in number within chimeric PVs

**Figure 3:**
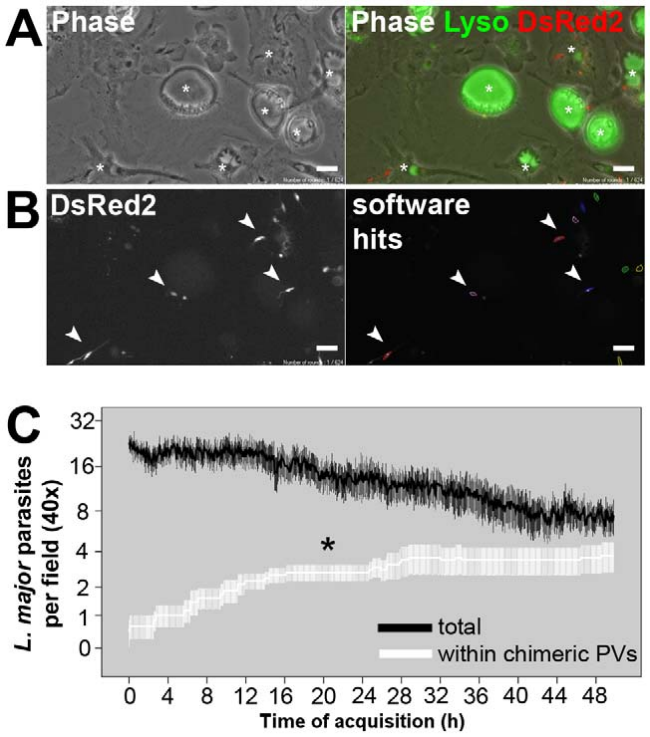
Algorithm-based recognition of *L. major*-DsRed2 parasites hosted by superinfected macrophages. (A) Example of an acquired field of macrophages infected for 48 hours with *L. amazonensis* and superinfected with *L. major*-DsRed2 metacyclic-enriched promastigotes. First picture is a phase contrast image (Ph2) acquired at 40x magnification, and second is the phase contrast image merged with RG fluorescence channels; Lysotracker in green, *L. major*-DsRed2 in red. *Leishmania amazonensis* PVs are indicated by asterisks. (B) Parasite recognition and quantification by Acapella software. The raw data (DsRed2 fluorescence) are shown in the first image and a quantified image is presented in the second (circles represent quantification hits). Examples of parasites recognized by the software are indicated by arrowheads. Bars = 10 µm. (C) Total number of *L. major*-DsRed2 parasites quantified by software algorithms (black line) and *L. major*-DsRed2 found within chimeric PVs (white line) quantified by time-lapse videomicrography observation. Acquisition started 2 hours after promastigote addition. Each line represents the mean quantification of 7 microscopic fields (40 x), at logarithmic (base 2) scale, plotted with s.e.m. The total number of promastigotes hosted by superinfected macrophages decreased after approximately 20 hours of experiment. There is a significant increase in the number of *L. major* parasites found within chimeric PVs in the first 12–20 hours of acquisition (One-way ANOVA with Tamhane's T2, Dunnett's T3 and Games-Howell Post Hoc multiple comparison tests between hourly time points, p<0.05).

The total number of *L. major*-DsRed2 promastigotes in superinfected macrophages was quantified by algorithm-based software analysis (Fig. 3). Images were taken from time-lapse recordings of 10 different microscopic fields, at 5 minutes intervals, and the total number of *L. major*-DsRed2 parasites per field were quantified at each time point (Fig. 3A–B). The number of *L. major* promastigotes inside chimeric PVs was accessed by direct observation of the time-lapse video records of each acquired microscopic field.

While the total number of *L. major* promastigotes fell, the number of *L. major*-DsRed2 promastigotes within chimeric PVs increased (Fig. 3C). At 12 hours of image acquisition (16 hours after *L. major*-DsRed2 promastigote addition), 13.6% (±4.68 s.e.m., n = 7) of promastigotes are found inside chimeric PVs. At 48 hours of acquisition, this percentage raised to 62.79% (±19.9 s.e.m., n = 7). The destruction of *L. major* promastigote took place exclusively in unfused donor PVs (data not shown). The accumulation of *L. major* promastigote within chimeric PVs was due to continuous transfer of *L. major* promastigotes to *L. amazonensis* PVs in the first 12 hours after superinfection.

### *Leishmania major* promastigotes multiplied within large, acidic *L. amazonensis* PVs

In the metacyclic-enriched bath of promastigotes administered to macrophages in superinfection, we expected a mixed population of *L. major* metacyclic and procyclic parasites, which contact and are transferred to *L. amazonensis* PVs. Thus, we investigated by live imaging if *L. major* promastigotes within chimeric PVs would be destroyed, behave like procyclic or stationary promastigotes or differentiate into amastigotes.

**Figure 4:**
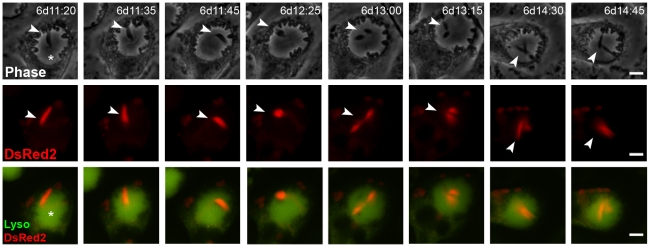
*L. major* promastigotes multiply inside chimeric PVs. Time-lapse recording of macrophages infected with *L. amazonensis*-WT for 48 hours and superinfected with metacyclic-enriched *L. major*-DsRed2 promastigotes. Image acquisition started 6 days after *L. major*-DsRed2 promastigote addition. Division of *L. major*-DsRed2 promastigote (arrowheads) inside *L. amazonensis*-WT PV (asterisk) was documented. The figure shows phase contrast (Ph2) in the first row, DsRed2 signal in the second, and Lysotracker merged with DsRed2 signal in the third. Time after promastigote addition is shown (d:h:min). Scale at 10 µm.

While some promastigotes did not divide, we often observed multiplication of *L. major* promastigotes within chimeric PVs. In Fig. 4 and Video S3, one *L. major*-DsRed2 promastigote (6 days after superinfection) was tracked within a large and acidic *L. amazonensis* PV. The promastigote moved freely in the chimeric vacuoles and kept the DsRed2 fluorescence, then displayed decreased movement and morphological changes at time point 11:45 h; at 12:25 h we identified two promastigote bodies bound by the parasite anterior pole. From time point 13:00 h, the promastigote completed a division, so we could observe two promastigotes moving inside *L. amazonensis* PV. The division occurred at stabilized temperature of 34°C as shown by temperature log of Nikon Biostation acquisition chamber (data not shown). Division of *L. major*-DsRed2 promastigotes within chimeric PVs was also observed in other series of images, from 24 hours to 96 hours after promastigotes addition (data not shown).

To investigate the influence of high temperature and low pH in promastigote multiplication, the growth curves of *L. major*-DsRed2 promastigotes axenically cultivated under different pH and temperature conditions were compared (Fig. S1). The growth of *L. major*-DsRed2 promastigote cultures at acidic pH was slower than that at neutral environment at 26°C (paradigmatically the optimal temperature for promastigote cultivation). Morphology and movement were preserved at 26°C, pH 5.0 or 7.2. At 34°C, *L. major*-DsRed2 promastigotes grew as well in media adjusted to pH 5.0 or 7.2, with no apparent difference for 3–4 days. After that time, at 34°C at pH 5.0 or 7.2, *L. major* promastigotes enter death phase, presenting altered morphology and DsRed2 emission, and presence of debris.

The growth kinetics of *L. major*-DsRed2 promastigotes within chimeric vacuoles was not directly demonstrated. However, in additional experiments, *L. major* promastigotes were isolated from coinfected macrophages after 3 or 5 days of superinfection with log-phase *L. major* promastigotes. The infectivity of the isolated parasites was tested on fresh macrophage cultures; it was found that *L. major* promastigotes isolated from coinfected macrophages were infective and that the infectivity was higher at 5 days than at 3 days of coinfection (unpublished data).

### Differentiation of *L. major* promastigotes into amastigotes did not take place within chimeric PVs

**Figure 5:**
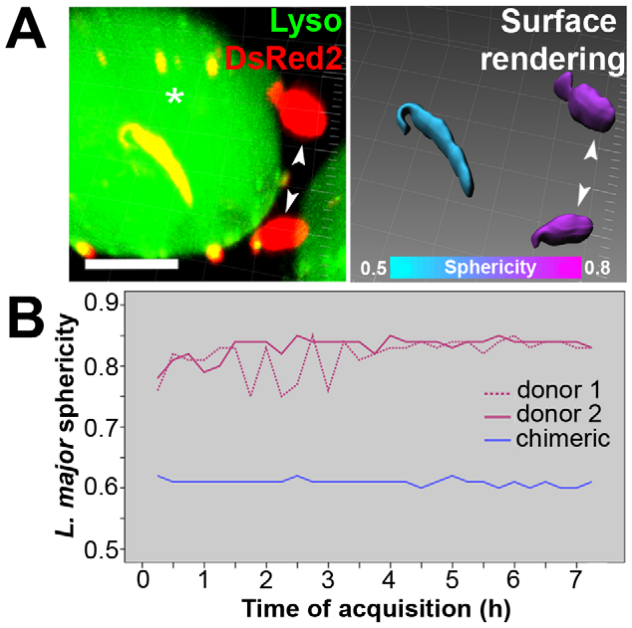
Multidimensional image of metacyclic-enriched *L. major*-DsRed2 promastigotes in superinfected macrophages. (A) On the left, multidimensional image of a chimeric PV (asterisk) and *L. major*-DsRed2 parasites sheltered by unfused donor PVs (arrowheads); Imaris MIP filter. On the right, surface rendering of parasites through DsRed2 channel allowed the software to assign a colorimetric scale to each *L. major-*DsRed2 parasite: it displays the sphericity parameter, ranging from cyan (less spherical, 0.5) to magenta (more spherical, 0.8); Imaris blend filter. Images were acquired 24 hours after addition of *L. major*-DsRed2 metacyclic-enriched promastigotes to macrophages. Bar = 10 µm. (B) Sphericity measurements during coinfection, presented by *L. major*-DsRed2 parasites hosted within unfused donor PVs (magenta lines) or within chimeric PV (blue line). Acquisition of multidimensional images started 12 hours after *L. major*-DsRed2 metacyclic-enriched promastigotes were added to macrophages.

We tracked *L. major-*DsRed2 metacyclic-enriched promastigotes hosted by superinfected macrophage and we observed differentiation into amastigotes forms exclusively within unfused donor PVs. We compared the morphology of *L. major* parasites within chimeric or donor PVs by multidimensional imaging (Fig. 5). Software surface rendering allowed measurement of parasite features such as volume and sphericity that were used as markers of promastigote-to-amastigote differentiation. Within donor vacuoles, the increase in sphericity and decrease in volume occurs in approximately 2 hours (data not shown). Within chimeric PVs, promastigotes maintained their initial volume and sphericity measurements, and displayed typical promastigote morphology, i.e., flagellated and elongated (Fig. 5A).

This site-dependent morphology was maintained for several hours in superinfected macrophages (Fig. 5C). *Leishmania major-*DsRed2 promastigotes in donor PVs presented a sphericity of 0.8–0.85 while promastigotes within chimeric PVs remained elongated (with sphericity near 0.6) and displayed flagellar movement. Non-dividing *L. major* promastigotes within chimeric PVs were tracked for 48 hours through live imaging; they kept flagellar movement and elongated morphology, with no apparent signs of differentiation (data not shown). Immunolabeling of LAMP1 in macrophages superinfected with *L. major* metacyclic-enriched promastigotes for 6 days revealed the presence of amastigotes exclusively in unfused membrane-bound PVs (data not shown).

## Discussion

We have shown that the PVs containing *L. major* amastigotes adhered to but did not fuse with preformed *L. amazonensis* spacious vacuoles. In contrast, *L. major* log phase promastigotes and promastigote suspensions enriched in metacyclic parasites were delivered by vacuolar fusion to *L. amazonensis* PVs. In the chimeric vacuoles thus formed, *L. major* promastigotes multiplied but did not differentiate into amastigotes, whereas in the same macrophages, *L. major* promastigotes sheltered in their own vacuoles, either died or differentiated into amastigotes. The biochemical and molecular mechanisms that underlie the lack of fusion of incoming *L. major* amastigote-containing PVs with *L. amazonensis* vacuoles and the permissiveness of the latter for fusion with *L. major* promastigote-carrying PVs, remain to be elucidated.

In contrast with the observation of fusion between intraspecific *L. amazonensis* PVs [20], the present results show that *L amazonensis* amastigote-PVs did not fuse with incoming PVs that contained *L. major* amastigotes (Fig. 1 and Video S1). In the coinfected cells, *L. major* PVs kept their usual morphology and the parasites multiplied as they did in monoinfected cells. Although Rab5 has been assumed to mediate homotypic fusion of early Rab5-positive *L. mexicana* PVs [13], we did not detect homotypical fusion between Rab7/LAMP1/LAMP2-positive PVs that sheltered *L. major* or *L. amazonensis* amastigotes. Thus, Rab7 may be responsible for the close contact observed between interspecific PVs, but their fusion may require additional factors.

Contrasting with the lack of fusion of *L. major* amastigotes-containing PVs with preformed *L. amazonensis* PVs, about 10% of incoming *L. major* promastigotes (from either log-phase or metacyclic-enriched populations) were found within *L. amazonensis* PVs. Multidimensional images allowed for the spatial visualization of fusion events in the first 12 hours after superinfection with promastigotes (Fig. 2–3 and Video S2). Thus, interspecific fusion of PVs is parasite-stage dependent.

Interspecific fusion was assumed to be regulated by parasite surface ligands, such as small size glycoconjugates [27], [28], [29], and/or by parasite-secreted macromolecules inserted in PV membranes and/or targeted to the cytosol [30]. The composition and fusogenicity of *Leishmania* PVs may also depend on the relative contribution to the PV membranes of the plasma membrane, endocytic and autophagic vesicles and/or endoplasmic reticulum [31].

Parasitophorous vacuoles of different *Leishmania* species have been isolated and compositional studies were initiated [32], [33], [34]. We believe that the characterization of macromolecules and other factors involved in fusion between PVs will require *in vitro* reconstitution of fusion of isolated *Leishmania* PVs [35], [36], [37], [38].

Rather surprisingly, *L. major* promastigotes that reached *L. amazonensis* PVs, instead of differentiating into amastigote stages or being destroyed by an acidic, phagolysosome-like environment, survived and multiplied while retaining the promastigote morphology and flagellar movement (Fig. 4 and Video S3). This unprecedented observation stands in marked contrast with the results of the intraspecific model, in which *Leishmania amazonensis* stationary-phase promastigotes survived, did not multiply and differentiated into amastigotes within PVs previously formed by the same species [20].

The extended survival of log-phase *L. major* procyclic promastigotes in chimeric PVs contrasts with the rapid destruction of log-phase promastigotes in their own, unfused vacuoles. It has been proposed that parasites sheltered in large vacuoles are protected from macrophage microbicidal effectors [26], [39], [40]. Additionally, proteophosphoglycans (PPG) secreted by *L. mexicana* amastigotes, were shown to attenuate macrophage leishmanicidal activity [27]. It is thus, conceivable that PPG secreted by *L. amazonensis* amastigotes could account for the survival of *L. major* log-phase promastigotes in chimeric PVs.

When metacyclic-enriched populations of *L. major* promastigotes were added to macrophages previously infected with *L. amazonensis*, differentiation of *L. major* was only observed within their own unfused, membrane-bound vacuoles (Fig. 5). Stationary-phase *L. major* promastigotes were found moving within chimeric PVs during time-lapse image acquisitions. The reasons for the lack of *L. major* differentiation are not understood. One possibility is that the pH within the *L. amazonensis* PVs is not optimal for differentiation of the *L. major* promastigotes; another is that such differentiation could require the close contact of the *L. major* parasites with their vacuolar membranes.

The interspecific PV fusion and transfer of *L. major* promastigotes into *L. amazonensis* PVs provides an additional answer to the recurrent question: “to what extent can a microorganism survive and multiply in vacuoles customized by a different pathogen?” There is, however, no reason to assume that a general answer will be necessarily found.

It has been assumed that genetic exchange, rarely found between *Leishmania* species, might take place in doubly infected vectors [41]. Patients infected with two different *Leishmania* species have been described in the literature [42]. If the present *in vitro* findings would mimic the *in vivo* situation, the isolation of amastigotes in parasite species-specific PVs could restrict the genetic exchange and *Leishmania* speciation to mixed populations of promastigotes in insect-vectors.

Finally, our results emphasize the usefulness of continuous live recordings in studies of intracellular parasitism [43]. It is hoped that additional experiments will map the fusogenicity of the spacious vacuoles of the *mexicana* group with each other and with other *Leishmania* species confined to small parasitophorous vacuoles.

## Supporting Information

Figure S1 Algorithm-based recognition of *L. major*-DsRed2 promastigotes axenically cultivated. (A) Parasite recognition and quantification by Image Pro Plus Software. The raw data are shown on the left (parasites DsRed2 fluorescence) and quantified image on the right (crosses represent quantification hits). Bars = 20 µm. (B–C) Growth curves of *L. major*-DsRed2 promastigotes at 34°C or 26°C, and pH 7.2 (B) or 5.0 (C). Parasites were counted by software per microscopic field and the numbers were normalized to a 10x field. Each line is representative of 10 microscopic fields per condition, with triplicates. Graphs are associated to images showing the morphological aspect of *L. major*-DsRed2 promastigotes after 4 and 10 days of cultivation at 34°C. Scale at 20 µm.
(0.47 MB TIF)

Video S1 Interaction between *L. amazonensis* vacuoles and *L. major* amastigote PVs. (A) Live imaging of macrophages previously infected with *L. amazonensis*-WT for 48 h and then superinfected with *L. major*-DsRed2 amastigotes. Video shows a macrophage, loaded with Lysotracker (green) and hosting the two species, at phase contrast and fluorescence merged channels. Image acquisition started at 72 hours after *L. major*-DsRed2 amastigote addition. Time after *L. major*-DsRed2 amastigote addition is shown (d:h:min:sec). Bar = 20 µm. (B) Live multidimensional imaging of macrophages previously infected with *L. amazonensis*-WT for 48 h and then superinfected with *L. major*-DsRed2 amastigotes. *Leishmania major*-DsRed2 amastigotes, sheltered by tight PV, weakly stained with Lysotracker (green), were observed at the periphery of large, Lysotracker-positive, *L. amazonensis*-WT PV. Multidimensional images were constructed using Imaris blend filter, and rotated in different angles. Time after *L. major*-DsRed2 amastigote addition is shown (d:h:min). Bar = 5 µm.
(2.18 MB MOV)

Video S2 Fusion between *L. amazonensis* and *L. major* PVs recorded by multidimensional imaging of macrophages previously infected with *L. amazonensis*-WT for 48 hours and then superinfected with *L. major*-DsRed2 metacyclic-enriched promastigotes. (A) *Leishmania major*-DsRed2 promastigote (red), sheltered within tight PV, weakly stained with Lysotracker (green), fused with large, Lysotracker-positive, *L. amazonensis*-WT PV. (B) Lysotracker channel, showing changes in PV shape and remodeling at the end of process. (C) Promastigote DsRed2 channel, showing the transfer of promastigote by parasite posterior pole. (D) Image rotation for visualization of fusion process from the bottom of the sample. Time after *L. major*-DsRed2 promastigote addition is shown (h:min). Multidimensional images were constructed using Imaris blend filter. Bar = 10 µm.
(0.82 MB MOV)

Video S3 Multiplication of *L. major*-DsRed2 promastigote within chimeric PV. Time-lapse recording of macrophages previously infected with *L. amazonensis*-WT for 48 hours and then superinfected with metacyclic-enriched *L. major*-DsRed2 promastigotes. Image acquisition started 6 days after *L. major*-DsRed2 promastigote addition. (A) Phase contrast (Ph2); (B) DsRed2 channel; (C) Lysotracker channel (green) merged with *L. major*-DsRed2 channel (red); (D) all channels merged. Time after promastigote addition is shown (d:h:min:sec). Bar = 10 µm.
(1.60 MB MOV)
