# Supplementary figures and images for: Fusion between Leishmania amazonensis and Leishmania major Parasitophorous Vacuoles: Live Imaging of Coinfected Macrophages
Source: PLoS Negl Trop Dis. 2010 Dec 7;4(12):e905. doi: 10.1371/journal.pntd.0000905 (PMC2998430; doi:10.1371/journal.pntd.0000905)

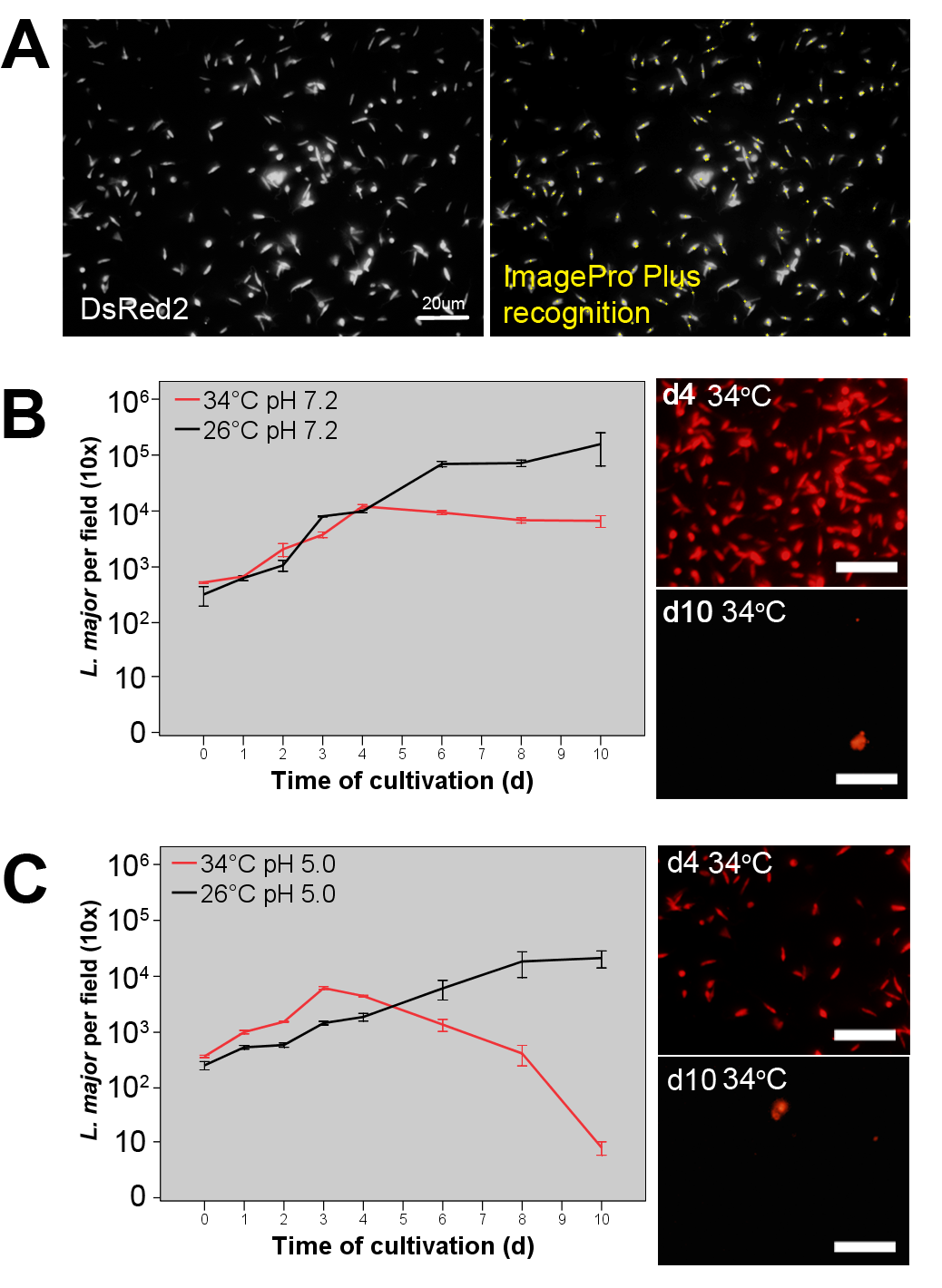

Supplement: Figure S1 — Algorithm-based recognition of L. major-DsRed2 promastigotes axenically cultivated. (A) Parasite recognition and quantification by Image Pro Plus Software. The raw data are shown on the left (parasites DsRed2 fluorescence) and quantified image on the right (crosses represent quantification hits). Bars = 20 µm. (B–C) Growth curves of L. major-DsRed2 promastigotes at 34°C or 26°C, and pH 7.2 (B) or 5.0 (C). Parasites were counted by software per microscopic field and the numbers were normalized to a 10x field. Each line is representative of 10 microscopic fields per condition, with triplicates. Graphs are associated to images showing the morphological aspect of L. major-DsRed2 promastigotes after 4 and 10 days of cultivation at 34°C. Scale at 20 µm. (0.47 MB TIF) [file pntd.0000905.s001.tif]
